# Un cas isolé de tuberculose appendiculaire

**DOI:** 10.4314/pamj.v9i1.71191

**Published:** 2011-06-07

**Authors:** Moujahid Mountassir, Issam Ennafae, Hicham Kechna, Samir Berrada, Siffedine El Kandry

**Affiliations:** 1 5^ème^ Hôpital Militaire Guelmim, Maroc

**Keywords:** Appendice, tuberculose extra pulmonaire, diagnostic, traitement

## Abstract

La tuberculose est une affection qui sévit à l’état endémique dans notre pays, elle demeure un problème majeur de santé publique .la tuberculose appendiculaire primitive est une affection très rare. Nous rapportons le cas d'un patient de sexe masculin âgé de 17 ans, admis au service pour une prise en charge d'une douleur de la fosse iliaque droite évoluant dans un contexte fébrile avec conservation de l’état général posant le diagnostic d'une appendicite aigue. Une appendicectomie a été réalisée ; le compte rendu anatomopathologique était en faveur d'une tuberculose appendiculaire isolée. Le patient a été mis sous traitement anti-bacillaire complémentaire pendant neuf mois avec une bonne évolution clinique. Le diagnostic de tuberculose doit être évoqué en premier surtout dans les pays d'endémie tuberculeuse.

## Introduction

La tuberculose reste une affection fréquente dans le monde; neuf millions de nouveaux cas et le nombre de décès annuels dû à la tuberculose est de 3 millions .Elle demeure un problème majeur de santé publique. Au Maroc durant l'année 2007, 500 à 1000 décès sont dus à la tuberculose, et 25562 nouveaux cas ont été répertoriés, ce qui correspond à un taux d'incidence de 87 nouveaux cas pour 100 000 habitants [1].C'est une affection qui peut toucher n'importe quel organe. La tuberculose appendiculaire primitive est une affection très rare. L'objectif de ce travail est de rapporter un cas de tuberculose appendiculaire isolée colligé dans le service de chirurgie au 5ème Hôpital Militaire de Guelmim qui reste une affection très rare.

## Observation

**Figure 1:**
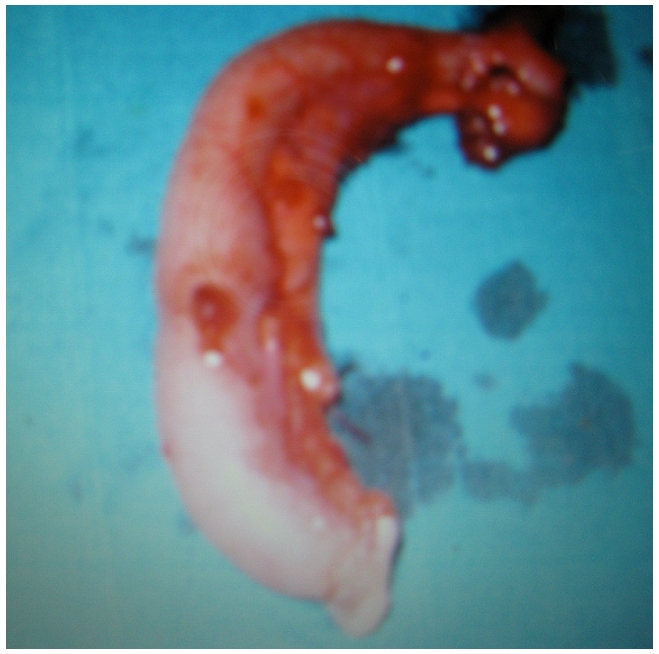
pièce opératoire d'appendicectomie chez un patient de 17 ans traité pour tuberculose appendiculaire

**Figure 2:**
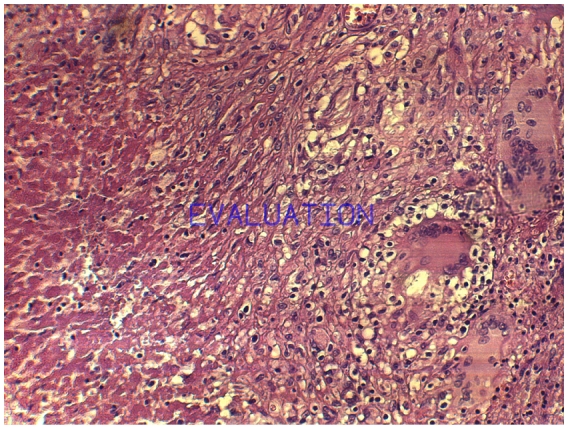
pièce opératoire d'appendicectomie, coupe histologique montrant un épithélium de revêtement régulier et une séreuse siège de granulomes épithélio-giganto-cellulaires centrés de nécrose caséeuse

**Figure 3:**
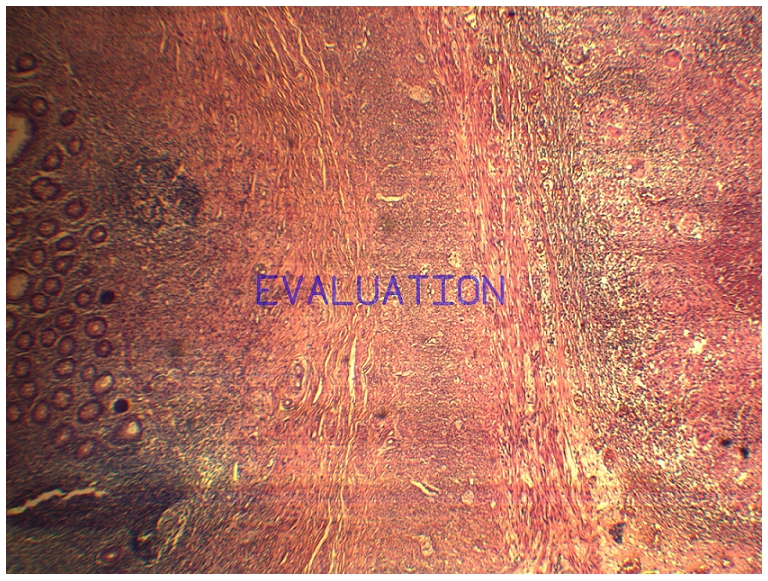
pièce opératoire d'appendicectomie, coupe histologique montrant l'appendice à gauche et le granulome à droite

Il s'agissait d'un patient âgé de 17 ans, sans antécédents médicaux chirurgicaux admis par le biais des urgences pour la prise en charge d'une appendicite aigue. L'examen clinique a montré une douleur de la fosse iliaque droite avec un signe de Mac Burney positif, évoluant dans un contexte fébrile à 38,5°C. Au bilan biologique une hyperleucocytose à 16000 éléments /mm^3^ et une CRP à 25. L’échographie abdominale a montré un aspect en faveur d'une appendicite sans signe d’épanchement intra péritonéal. Le patient a été opéré par une voie cælioscopique, l'exploration abdominale a montré un appendice inflammatoire en position latéro-caecale sans signe d’épanchement. Le mésentère, la région iléo-caecale, l'iléon, et le reste du tube digestif ne présentaient aucune lésion macroscopiquement décelable en dehors de l'aspect inflammatoire de l'appendice. Nous avons réalisé une simple appendicectomie (Figure 1). Les suites post opératoires étaient simples et le patient a quitté le service au 3ème jour. Le compte rendu anatomopathologique a montré un épithélium de revêtement régulier avec une séreuse qui est le siège de granulomes épithélio-giganto-cellulaires centrés de nécrose caséeuse ce qui était en faveur d'une tuberculose appendiculaire isolée (Figure 2 et Figure 3).Le patient a été confié au service de médecine interne pour bilan et prise en charge, la recherche de Bacille de Koch (BK) dans les crachats et les urines était négative de même que l'intradermoréaction à la tuberculine. Un traitement antituberculeux à base de streptomycine, rifampicine, isoniazide et pyrazinamide pendant sept mois suivi d'une association à base de rifampicine et isoniazide pendant deux mois a été instauré avec une bonne évolution clinique et biologique.

## Discussion

La tuberculose est une maladie de l'adulte jeune: 70% des malades sont âgés de 15 à 45 ans [2].La contamination inter humaine se fait par voie aérienne le plus souvent à partir de gouttelettes de salive, soit par une diffusion hématogène. Les autres voies de contamination muqueuse, digestive, cutanée sont possibles mais rares [3]. L′atteinte la plus fréquente du tube digestif est représentée par la région iléo-caecale car elle est abondante en tissu lymphoïde et représente également une zone de stase relative [4]. Les localisations péritonéales de la tuberculose sont rares mais classiques [5]. L′atteinte appendiculaire lors de la tuberculose est très rarement révélatrice de la maladie. Elle serait retrouvée dans 0,26% à 2,3% des séries d'appendicectomies des patients porteurs d'une tuberculose maladie [6].Elle peut être rencontrée lors de la présence d′une miliaire tuberculeuse multi viscérale ou chez des patients immunodéprimés [5]. La pathogenèse de la tuberculose appendiculaire n'est pas très bien comprise mais trois voies de contamination possibles sont retenues : digestive (la plus fréquente), hématogène et par contiguïté [7]. Trois formes anatomo-cliniques sont décrites [8]. La forme chronique faite de vagues douleurs abdominales, évoluant par crises successives, associées à des vomissements, inconstants, et à de la diarrhée. Cette forme est le plus souvent confondue à la tuberculose iléo-cæcale. La forme aiguë présente une symptomatologie caractéristique de l'appendicite aiguë phlegmoneuse. Dans la forme dite latente, l'appendice paraît normal à la macroscopie, le diagnostic de tuberculose appendiculaire étant de découverte fortuite à l'examen histologique. Le diagnostic peropératoire de la tuberculose isolée de l'appendice est difficile, voire quasi-impossible [9] du fait de la rareté de cette localisation et de la diversité des formes anatomo-cliniques. L'aspect macroscopique [10] distingue des formes hypertrophiques d'aspect pseudo tumoral et des formes ulcéreuses responsables d'abcès et de perforations mais l'appendice peut être normal. Sur le plan biologique, il existe fréquemment un syndrome inflammatoire. L′intradermoréaction à la tuberculine n′est ni sensible, ni spécifique. L'examen histologique apporte la certitude du diagnostic devant la présence du follicule de Koester qui correspond à un granulome épithélioïde et gigantocellulaire centré par une nécrose caséeuse et délimité par une couronne lymphocytaire (Figure 1). Chez les personnes immunodéprimés, les formes exsudatives et nécrosantes sont plus fréquentes que les formes granulomateuses, nécessitant ainsi une coloration de Ziehl pour la mise en évidence du bacille de Koch et afin d'en faire le diagnostic différentiel avec une actinomycose, une histoplasmose, une yersiniose ou une maladie de Crohn [6, 8].

La symptomatologie clinique est celle d'une crise appendiculaire classique avec une douleur de la fosse iliaque droite dans un contexte fébrile avec altération de l’état général. Dans sa forme primitive, très rare, elle réalise des aspects souvent trompeurs, de diagnostic difficile. Sur le plan biologique, il existe fréquemment un syndrome inflammatoire. L′intra dermo-réaction à la tuberculine n′est ni sensible, ni spécifique. Le bilan hépatique est parfois perturbé. Dans notre cas l'examen clinique a montré une douleur de la fosse iliaque droite évoluant dans un contexte fébrile à 38,5°C et un signe de Murphy positif. Le bilan biologique a montré une hyperleucocytose à 16000 éléments /mm^3^, une CRP à 25. La radiographie pulmonaire est d′un grand intérêt quand elle montre des lésions de tuberculose pleuro pulmonaire évolutive ou séquellaire.

Le traitement de la tuberculose appendiculaire est habituellement médical se basant sur des antituberculeux (Streptomycine, Rifampicine, Isoniazide, Pyrazinamide) pendant deux mois et Rifampicine, isoniazide pendant quatre mois. Certains auteurs préconisent jusqu′à 9 mois de traitement voire une année [3, 8, 10]. L′évolution est favorable dans la majorité des cas (90%) avec disparition des symptômes [7, 10]. Le recours à la chirurgie se fait à visée thérapeutique, pour réaliser une appendicectomie et éradiquer le foyer inflammatoire ou en cas de complication. Dans notre cas, le diagnostic a été réalisé lors d'une appendicectomie sur la pièce opératoire.

## Conclusion

La tuberculose appendiculaire représente environ 1% de l'ensemble des tuberculoses digestives. Son diagnostic doit toujours être évoqué surtout dans les pays d'endémie tuberculeuse. Le traitement est surtout chirurgical associé au traitement médical antituberculeux.
